# 1770. Rickettsial Disease Outbreak in Northeastern Mexico: A 15-Month Epidemiological Analysis

**DOI:** 10.1093/ofid/ofad500.1600

**Published:** 2023-11-27

**Authors:** Ricardo J Estrada-Mendizabal, Oscar Tamez-Rivera, Emelina Hinojosa Vela, Paulina Blanco Murillo, Cordelia Alanis-Garza, Jaime Flores-Gouyonnet, Jessica Suhail Sauceda Garza, Gloria Yolanda Carranza Medina, Edgar Paolo Rodriguez Vidales, Lilia Elida Garcia Rodriguez, Alma Rosa Marroquin Escamilla

**Affiliations:** Tecnologico de Monterrey, Escuela de Medicina y Ciencias de la Salud, Monterrey, Nuevo Leon, Mexico; Tecnologico de Monterrey, Escuela de Medicina y Ciencias de la Salud, Monterrey, Nuevo Leon, Mexico; Secretaria de Salud de Nuevo Leon, Monterrey, Nuevo Leon, Mexico; Tecnologico de Monterrey, Escuela de Medicina y Ciencias de la Salud, Monterrey, Nuevo Leon, Mexico; Tecnologico de Monterrey, Escuela de Medicina y Ciencias de la Salud, Monterrey, Nuevo Leon, Mexico; Universidad Autonoma de San Luis Potosi, Facultad de Medicina, San Luis Potosi, San Luis Potosi, Mexico; Secretaria de Salud de Nuevo Leon, Monterrey, Nuevo Leon, Mexico; Secretaria de Salud de Nuevo Leon, Monterrey, Nuevo Leon, Mexico; Secretaria de Salud de Nuevo Leon, Monterrey, Nuevo Leon, Mexico; Secretaria de Salud de Nuevo Leon, Monterrey, Nuevo Leon, Mexico; Secretaria de Salud de Nuevo Leon, Monterrey, Nuevo Leon, Mexico

## Abstract

**Background:**

An unprecedented rise in rickettsiosis cases is occurring in Nuevo Leon (NL), Mexico. A total of 73 cases have been identified since Jan 2022, as opposed to 2021, when 13 cases occurred. Despite multidisciplinary efforts, the current outbreak is still ongoing. Early treatment with doxycycline is crucial to avoid devastating outcomes.

Figure 1

**Figure:**
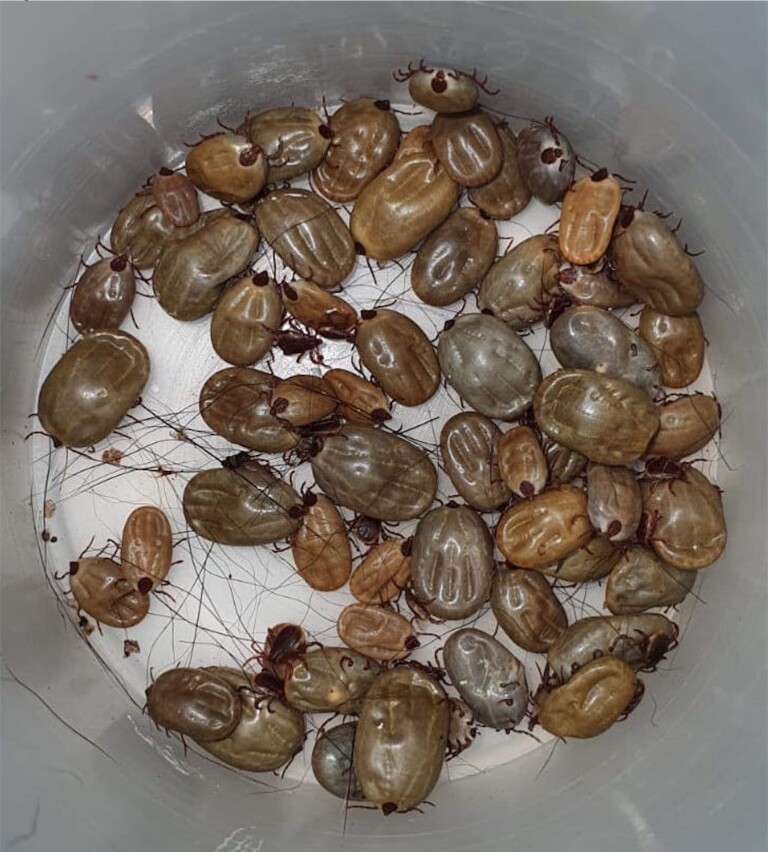

Brown-dog ticks collected by the vector control department of Nuevo Leon.

Figure 2

**Figure:**
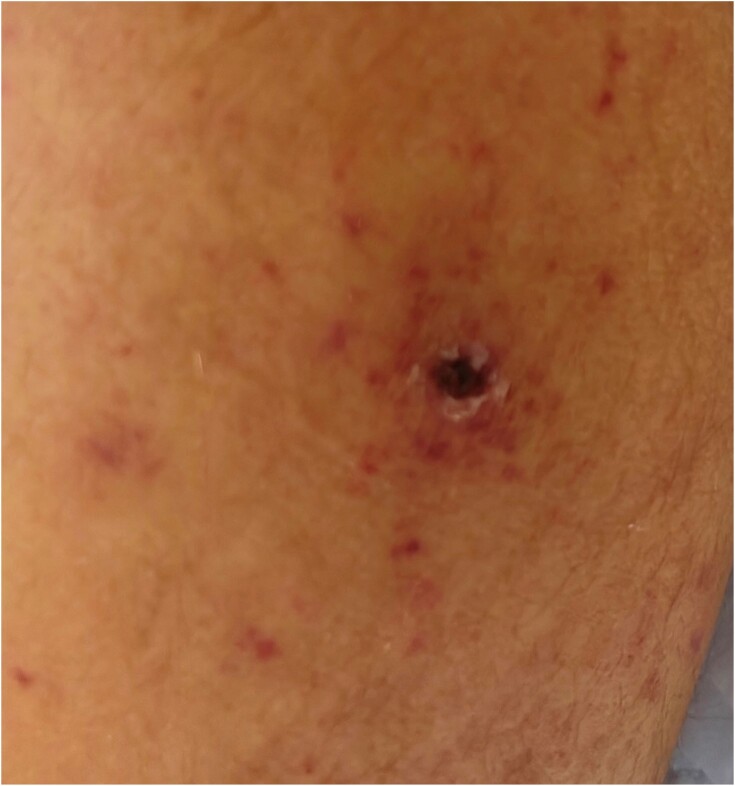

Inoculation eschar on the left leg of a patient infected with Rickettsia rickettsii.

**Methods:**

An ambispective, descriptive study was conducted to evaluate the demographic, clinical, and laboratory characteristics of patients living in NL who had a confirmed diagnosis of rickettsiosis from Jan 2022 to March 2023. Cases were confirmed through a positive real-time polymerase chain reaction (RT-PCR) followed by whole-genome sequencing (WGS) or seroconversion by indirect immunofluorescence antibody (IFA) assay. A chi-square test was performed to determine associations with mortality.

Figure 3.

**Figure:**
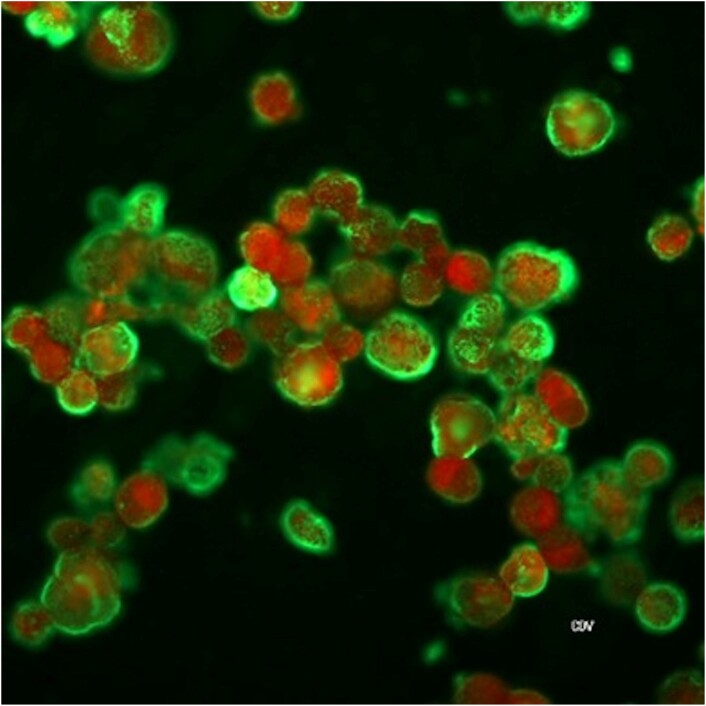

Indirect immunofluorescence antibody (IFA) assay with green fluorescence indicating the presence of Rickettsia on the sample.

**Results:**

In the studied period, 73 cases of rickettsiosis have been confirmed. This represents an incidence of 1.2 cases/100,000 inhabitants (compared to 0.2/100,000 in 2021). The median age was 11 years, and 57.5% were female. Most patients (90%) required hospitalization, and all had a positive history of tick exposure within two weeks before symptom onset. *R. rickettsii* was the predominant species (93.1%), followed by *R. typhi* (6.8%). The most frequent signs and symptoms were fever (100%), headache (77%), and abdominal pain (71%). Thrombocytopenia was present in 98%, and anemia in 58% of the cases. Fifty-two patients were treated with doxycycline. The median time-to-treatment initiation (TTI) from symptom onset was 4 days. High mortality (63%) was documented. Treatment with doxycycline (p=0.05) and a TTI of ≤ 24 hr (p=0.01) were associated with survival.

**Figure:**
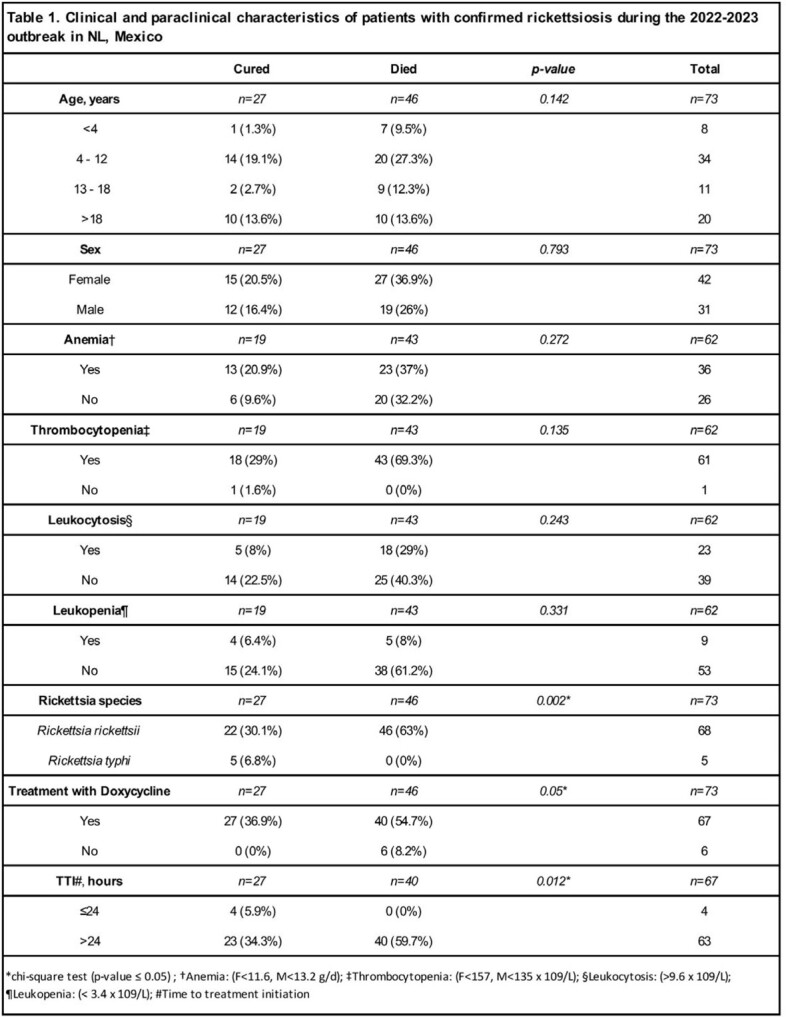

**Figure:**
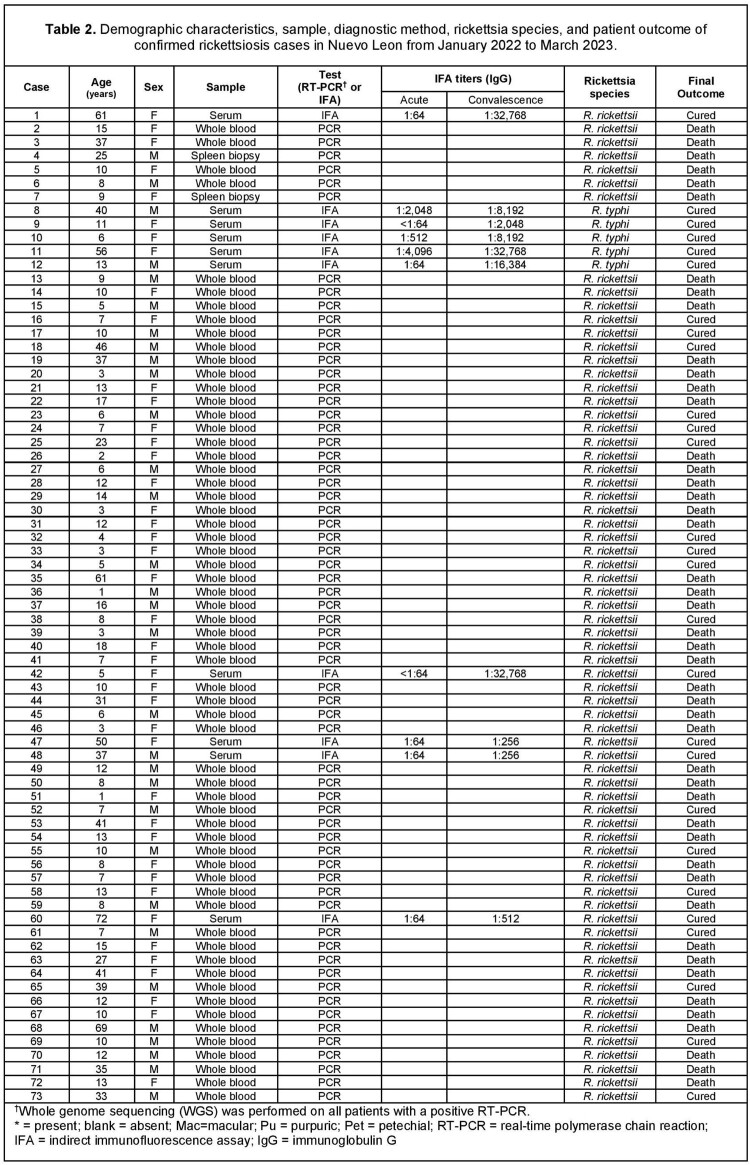

Figure 3.

**Figure:**
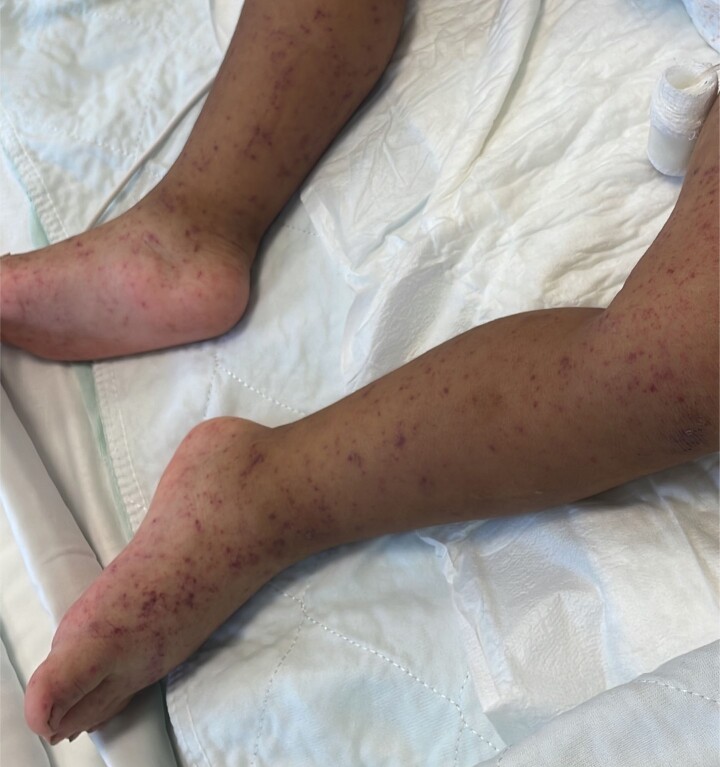

Petechial rash on a patient infected with Rickettsia rickettsii.

**Conclusion:**

The current outbreak of rickettsiosis in NL has conferred high morbidity and mortality, mainly in children. The fatality rate (63%) is alarming. Efforts have been made to control the outbreak by establishing vector control strategies, educating healthcare personnel, designating community champions against rickettsioses, and raising public awareness campaigns. Clinicians on the Mexico-United States border should have a high suspicion index of rickettsiosis among patients with clinical signs compatible with the disease and consider early antibiotic treatment to reduce mortality risk.

Figure 4.

**Figure:**
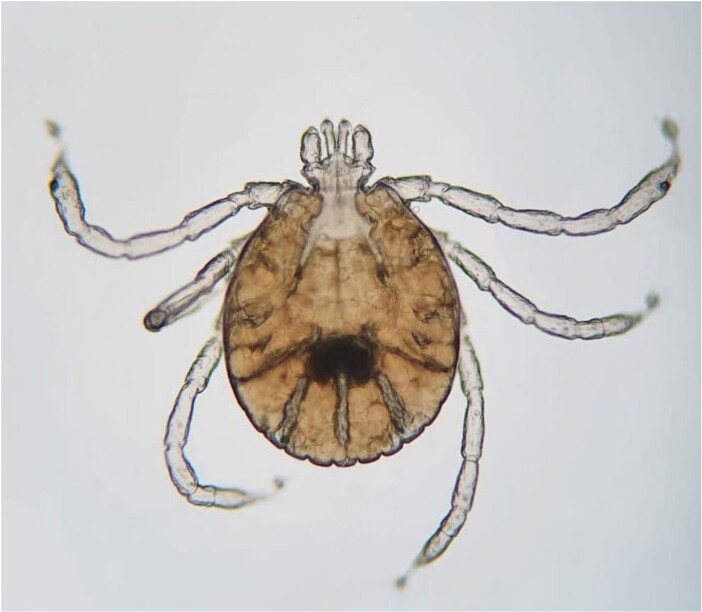

Brown-dog tick larva under the microscope.

**Disclosures:**

**All Authors**: No reported disclosures

